# Cationic Amino Acid Uptake Constitutes a Metabolic Regulation Mechanism and Occurs in the Flagellar Pocket of *Trypanosoma cruzi*

**DOI:** 10.1371/journal.pone.0032760

**Published:** 2012-02-29

**Authors:** Mariana R. Miranda, Melisa Sayé, León A. Bouvier, María de los Milagros Cámara, Javier Montserrat, Claudio A. Pereira

**Affiliations:** 1 Laboratorio de Biología Molecular de Trypanosoma cruzi (LBMTC), Instituto de Investigaciones Médicas “Alfredo Lanari”, Universidad de Buenos Aires and CONICET, Buenos Aires, Argentina; 2 Instituto de Investigaciones en Ingeniería Genética y Biología Molecular (INGEBI), Buenos Aires, Argentina; Federal University of São Paulo, Brazil

## Abstract

Trypanosomatids' amino acid permeases are key proteins in parasite metabolism since they participate in the adaptation of parasites to different environments. Here, we report that TcAAP3, a member of a *Trypanosoma cruzi* multigene family of permeases, is a *bona fide* arginine transporter. Most higher eukaryotic cells incorporate cationic amino acids through a single transporter. In contrast, *T. cruzi* can recognize and transport cationic amino acids by mono-specific permeases since a 100-fold molar excess of lysine could not affect the arginine transport in parasites that over-express the arginine permease (TcAAP3 epimastigotes). In order to test if the permease activity regulates downstream processes of the arginine metabolism, the expression of the single *T. cruzi* enzyme that uses arginine as substrate, arginine kinase, was evaluated in TcAAP3 epimastigotes. In this parasite model, intracellular arginine concentration increases 4-folds and ATP level remains constant until cultures reach the stationary phase of growth, with decreases of about 6-folds in respect to the controls. Interestingly, Western Blot analysis demonstrated that arginine kinase is significantly down-regulated during the stationary phase of growth in TcAAP3 epimastigotes. This decrease could represent a compensatory mechanism for the increase in ATP consumption as a consequence of the displacement of the reaction equilibrium of arginine kinase, when the intracellular arginine concentration augments and the glucose from the medium is exhausted. Using immunofluorescence techniques we also determined that TcAAP3 and the specific lysine transporter TcAAP7 co-localize in a specialized region of the plasma membrane named flagellar pocket, staining a single locus close to the flagellar pocket collar. Taken together these data suggest that arginine transport is closely related to arginine metabolism and cell energy balance. The clinical relevance of studying trypanosomatids' permeases relies on the possibility of using these molecules as a route of entry of therapeutic drugs.

## Introduction

*Trypanosoma cruzi* is the causative agent of Chagas ‘disease, a zoonosis affecting approximately 18 million people in the Americas [1]. This protozoan parasite has a complex life cycle, involving morphological changes and a wide variety of environments with different composition, mainly the insect vector gut, mammalian blood and mammalian host cell cytoplasm. In consequence, the parasite survival mainly depends on their adaptive ability and metabolic plasticity. A critical aspect to achieve these adaptations is the parasite potential to take advantage of the available metabolites in the different extracellular milieus using a large repertoire of nutrient permeases. One of the major amino acid transporter families is the “Amino Acid/Auxin Permease Family” (AAAP; TC 2.A.18) which is largely represented in plants [2]. In *T. cruzi*, many members of this family were first identified by our group [3] and then confirmed by the TriTryps genome project [4]. This *T. cruzi* family, called TcAAAP, has more than 30 genes coding for proteins with lengths of 400–500 amino acids and 10–12 predicted transmembrane α-helical spanners. One interesting feature of this permease family is the absence of similar sequences in mammalian genomes; however, the presence of unidentified orthologs could not be discarded [5]. *T. cruzi* arginine transport systems have been largely studied during the last decade [6]; even so, the first evidence of the molecular determinants of this process was recently reported. TcAAP3 (formerly TcAAAP411), a member of the TcAAAP family, has an arginine transport activity in a yeast model [7]. A similar arginine transporter (LdAAP3) was also identified in the protozoan parasite *Leishmania donovani* [8]. LdAAP3 regulation mainly depends on the availability of the extracellular substrate since amino acid starvation produces an increase in arginine transport and LdAAP3 protein abundance [9]. Interestingly, a similar mechanism of regulation was described for arginine and other *T. cruzi* amino acids transport systems [10], supporting the hypothesis that most of the previously characterized transport systems in trypanosomatids involve members of the TcAAAP family. On the other hand, lysine transporters from this family were also identified and characterized in *Trypanosoma* spp. and *Leishmania* spp. [11]. In higher eukaryotes most of cationic amino acids transporters incorporate arginine and lysine by a single permease [12], [13], [14]. On the contrary, parasite permeases translocate cationic amino acids by different mono-specific transporters [6], [11]. Taken together, these differences suggest that amino acid transporters may provide multiple and unexplored targets and gateways for therapeutic drugs.

In this work we validated the functionality of TcAAP3 in vivo by homologous over-expression in *T. cruzi* epimastigotes. We also studied the TcAAP3 specific localization in the parasite surface and its contribution on the regulation of arginine metabolism and cell energy balance.

## Materials and Methods

### Cell cultures

Epimastigotes of the MJ-Levin strain were cultured at 28°C in plastic flasks (25 cm^2^), containing 5 mL of LIT medium (started with 10^6^ cells per milliliter) supplemented with 10% fetal calf serum, 100 U/mL penicillin, and 100 µg/mL streptomycin [15]. The parasites were subcultured with passages each 7 days. Cells were counted using a hemocytometer. The MJ-Levin *T. cruzi* strain was recently typified according to the mini-exon sequences from twenty independent clones (Schijman et al., *personal communication*). Based on these results the strain has been included in the typing unit 1 [16].

### Arginine and lysine transport assays

Aliquots of epimastigote cultures (3×10^7^ parasites) were centrifuged at 8,000×g for 30 s, and washed once with phosphate-buffered saline (PBS). Cells were resuspended in 0.1 mL PBS and then added 0.1 mL of the transport mixture containing 100 µM L-(^3^H) arginine or lysine (PerkinElmer's NEN® Radiochemicals; 0.4 µCi). Following incubation for 10 min at 28°C, reaction was stopped by adding 1 mL of ice-cold PBS. Cells were centrifuged as indicated above, and washed twice with ice-cold PBS. Cell pellets were resuspended in 0.2 mL of water and counted for radioactivity in UltimaGold XR liquid scintillation cocktail (Packard Instrument Co., Meridien CT, USA) [6]. Assays were run at least by triplicate. Cell viability was assessed by direct microscopic examination. Non-specific uptake and carry over were measured in transport mixture containing 10 mM L-arginine (100-fold molar excess), or in standard transport mixture incubated at 4°C.

### Plasmid constructions and parasite tranfection

TcAAP3, TcBilbo1 and TcAAP7 genes (GeneDB: Tc00.1047053511411.30, Tc00.1047053511127.20, and Tc00.1047053511127.20, respectively) were amplified using genomic *T. cruzi* DNA as template and the following primers: TcAAP3F 5′ ATGGGCACCGAGAGTGGCAA 3′; TcAAP3R 5′ TTACCGAACCACACCATACA 3′; TcBilbo1F 5′ ATGTTGGTCATTAATGTAGCCGCTG 3′; TcBilbo1R 5′ GGATCCGGATCGTCCTCCCTGCAGCT 3′; TcAAP7F 5′ ATGTATGACAACGTCAATGAGG 3′; and TcAAP7R 5′ GTCGACTCAGCCATGGGCTTCG 3′. Amplification products were cloned into a modified pTREX expression plasmid called pTREXL [17]; (Bouvier *et al. unpublished observations*) or fused to mCherry (mCherry::TcAAP3; the name corresponds to the order in which the genes were fused) and eGFP (TcBilbo1::eGFP; eGFP::TcAAP7) genes present in the pTREXL plasmids. Constructions were transfected into *T. cruzi* epimastigotes as follows. 10^8^ parasites grown at 28°C in LIT medium were harvested by centrifugation, washed with PBS, and resuspended in 0.35 mL of electroporation buffer (PBS containing 0.5 mM MgCl_2_ and 0.1 mM CaCl_2_). This cell suspension was mixed with 50 µg of plasmid DNA in 0.2 cm gap cuvettes (Bio-Rad Laboratories). The parasites were electroporated using a single pulse of (400 V, 500 µF) with a time constant of about 5 ms.

### Fluorescence Microscopy

Epimastigote samples from the days 1–7 after transfection, were washed twice with PBS. After letting the cells settle for 30 min at room temperature onto poly-L-lysine coated coverslips, parasites were fixed at room temperature for 20 min with 4% formaldehyde in PBS, followed by a cold methanol treatment for 5 min. Slides were mounted using Vectashield with DAPI (Vector Laboratories). Cells were observed in an Olympus BX60 fluorescence microscope. Images were recorded with an Olympus XM10 camera.

### Amino acid and ATP determinations

Epimastigote cells were counted using a hemocytometric chamber, harvested by centrifugation at 1,500×g for 10 min and washed three times with PBS. Cell pellets were then resuspended in MilliQ water and lysed by 5 cycles of freezing and thawing. Samples (0.2 mL) were centrifugated and the soluble fraction was mixed with (5 µL) β-mercaptoethanol and allowed to stand for 5 minutes at room temperature followed by precipitation with ice-cold methanol (800 µL) while vortexing. Tubes were allowed to stand for 15 minutes in ice before centrifuging and the supernatant was collected. Efficiency of protein precipitation step was assessed by Bradford's method. The protein free supernatants obtained from 5×10^8^ parasites were dried in a rotary evaporator, resuspended in 20 µL of milliQ water and processed immediately for assaying total amino acids by HPLC analysis or stored at −80°C until further analysis. Amino acids were derivatizated and analyzed using the AccQ. Tag Amino Acid Analysis Method (Waters) according to the manufacturer instructions. ATP determinations were performed using the “ATP Bioluminiscence Assay Kit HS II” (Roche) according to the manufacturer instructions. Glucose determinations were performed using the “Enzymatic Glycemia Kit” (Biosystems) according to the manufacturer instructions. Assays were run at least by triplicate.

### Bioinformatics

Sequences from the “Tritryps” genome projects were obtained at GeneDB (http://www.genedb.org/) and TcruziDB (http://tcruzidb.org/). Assembly and analysis of the DNA sequence data were carried out using the software package Vector NTI v. 10.3.0 (Invitrogen) and the online version of BLAST at the NCBI (http://www.ncbi.nlm.nih.gov/BLAST/). Local or online software were used under default parameters. Reference metabolic pathways were obtained from the Kyoto Encyclopedia of Genes and Genomes - KEGG (www.genome.jp/kegg/).

## Results and Discussion

### TcAAP3 encodes an arginine transporter

**Figure 1:**
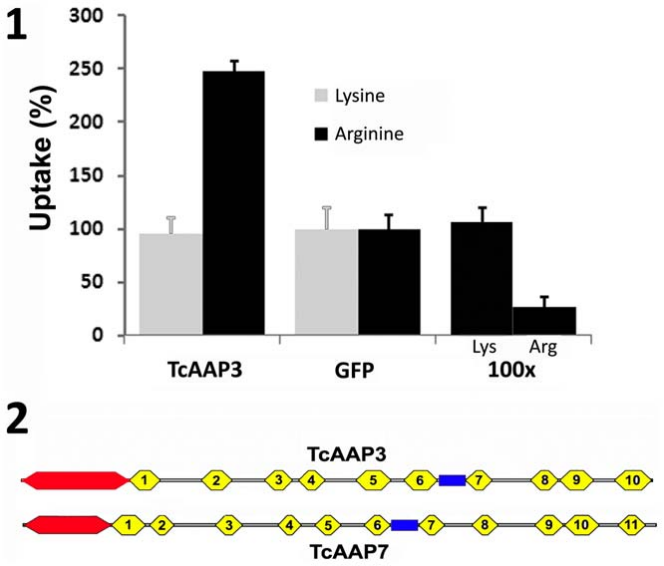
Substrate specificity of TcAAP3. 1) Radiolabeled L-arginine (100 µM, black) or L-lysine (100 µM, grey) uptake was measured in TcAAP3 over-expressing parasites (TcAAP3), GFP controls (GFP), and in the presence of 100-fold molar excess of lysine (Lys) or arginine (Arg). Uptake rates of arginine and lysine are expressed as a percentage of the GFP controls and correspond to 93 and 21 pmol/min per 10^8^ cells, respectively. 2) Schematic representation of the arginine permease (TcAAP3) and the previously characterized lysine permease (TcAAP7). N-terminal red diamonds represent the variable region of the TcAAAP permeases, numbered yellow diamonds represents the transmembrane spans, and blue boxes are the putative cationic amino acids binding motifs.

To study whether TcAAP3 is a *bona fide* arginine permease in *T. cruzi*, the full-length TcAAP3 and the eGFP genes were cloned in the expression vector pTREXL and epimastigote cells were transfected with the different plasmid constructions. After the selection period, pTREXL-TcAAP3 transfected parasites (TcAAP3 epimastigotes) showed an arginine transport rate of about 2.5-folds greater than pTREXL-eGFP controls (Figure 1, image 1). The increase in the arginine transport rate seems not to be well tolerated by *T. cruzi* epimastigotes, in contrast to the previously studied lysine transporter TcAAP7. While the transport of lysine has been increased in 50-folds by over-expression of TcAAP7 [11], in the case of arginine transport we failed to obtained cell clones exceeding about 2.5-folds the control rate using exactly the same approach. This phenomenon could be related to alterations in the *T. cruzi* arginine metabolism, in contrast to the lysine one, which is probably limited to osmoregulation and protein synthesis [11].

TcAAP3 epimastigotes did not present morphological differences respect to the controls. However, transfected parasites showed impaired growth kinetics, reaching a cell density of 7.5×10^7^ (±1.2×10^7^) cells per mL, and the GFP controls 12.3×10^7^ (±1.2×10^7^) cells per mL at the late stationary phase of culture. This growth phenotype could be due to the toxicity of the over-expressed membrane protein per se or to the metabolic effects as a consequence of increased arginine concentrations, as explained below.

### TcAAP3 is highly specific for arginine

Mammalian cells transporters incorporate arginine and lysine together through a single cation amino acid transporter; however, previous studies from our group demonstrated that the *T. cruzi* and *Leishmania* lysine permeases (AAP7) are mono-specific [11]. To test if TcAAP3 is also a mono-specific permease, arginine and lysine transport assays were performed using TcAAP3 epimastigotes and GFP transfected parasites. As Figure 1 (image 1) shows, TcAAP3 epimastigotes presented an increase only in arginine, but not in lysine transport rate compared to the pTREXL-GFP controls. Additionally, when arginine transport assays were performed in the presence of a 100-folds molar excess of lysine, the measured rates remain unaltered. As expected, only an excess of arginine produced an inhibition of the (^3^H)-L-arginine intake by isotopic dilution. These results confirm that TcAAP3 is, unlike mammalian transporters, a mono-specific arginine permease.

In order to predict the putative molecular determinants of the substrate specificity, a comparative bioinformatic analysis was performed using different approaches. The amino acid sequences of all members of the TcAAAP family present a highly variable N-terminal domain (about 90 amino acids, 5% of consensus) which constitutes a candidate to be the determinant of the permeases specificity [7]. Recently it was reported that these variable regions are phosphorilated by unidentified kinases that may represent a regulation mechanism of permeases activity [18]. In contrast, the central and C-terminal domains have >70% of consensus amino acid positions. Despite the variable regions of *T. cruzi* and *Leishmania* orthologs do not share any clearly similar motif, a region of negatively charged amino acids was found between the 6^th^ and the 7^th^ predicted transmembrane spans, corresponding to TcAAP3 residues 291–305 (Figure 1, image 2). This charged region, also present in TcAAP7 and in both TcAAP3 and TcAAP7 *Leishmania* orthologs, can be the cationic amino acids recognition motif.

### Over-expression of TcAAP3 increases the intracellular arginine concentration

In order to test if the elevated rates of arginine transport, derived from TcAAP3 over-expression, produce an increase in the intracellular arginine pools, amino acid concentrations were determined by derivatization and further resolution by reversed-phase chromatography. Control parasites transfected with an empty plasmid presented an arginine concentration of 0.46 mM and TcAAP3 epimastigotes 1.73 mM, representing an increase in intracellular arginine concentration of about 3.8-fold. No differences were observed in lysine concentration between both transfected parasite groups. These results not only reinforce the data about the mono-specificity of the transporter, but also suggest that if exist any compensatory mechanism that maintain the physiological levels of arginine, it is inactive or insufficient to restore the equilibrium in this parasite model.

### Arginine kinase is the only predicted arginine consuming enzyme in Trypanosoma cruzi

**Figure 2:**
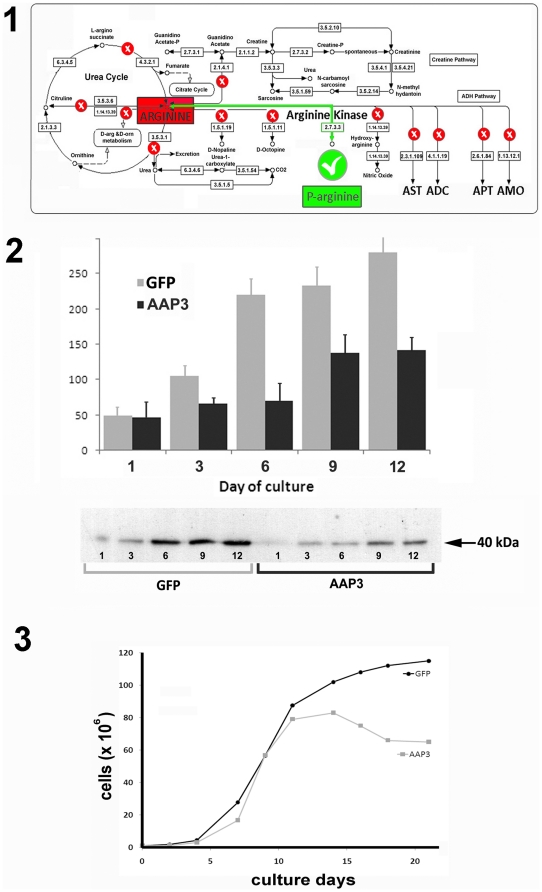
Arginine metabolism in *T. cruzi*. 1) Bioinformatic prediction of arginine metabolism. Using the KEGG arginine and proline metabolic pathways as a reference, genes coding for putative arginine metabolism enzymes in *T. cruzi* were searched using protein sequences from other organisms as baits. All enzyme EC numbers are indicated into the boxes, routes marked with a red cross were not identified in *T. cruzi*. AST: arginine N-succinyltransferase; ADC: arginine decarboxylase; APT: arginine-pyruvate transaminase; and AMO: arginine 2-monooxygenase. 2) Western blot analysis of arginine kinase expression was performed along the parasite growth curve between days 1 and 12. GFP control parasites (GFP) and TcAAP3 over-expressing parasites (AAP3) were compared by band densitometry (upper panel). Arrow indicates the position of the 40 kDa molecular weight marker. 3) A comparative epimastigotes growth curve was calculated from control (GFP) and TcAAP3 (AAP3) parasite cultures during 21 days.

Arginine metabolism has been largely studied in trypanosomes but there are still many unresolved issues. *T. cruzi* is unable to synthesize arginine; therefore the amino acid is obtained from the host through different transport systems [19]. In trypanosomatids, most of the well-studied enzymatic reactions involving L-arginine have been related to the ornithine-arginine pathway. *T. cruzi* lack ornithine decarboxylase, arginine decarboxylase and arginase, indicating that it is unable to neither synthesize diamines from either L-arginine or L-ornithine, nor excrete the nitrogenous waste by the urea cycle [19]. To identify genes coding for arginine metabolizing enzymes, a carefully bioinformatic approach by data mining was performed. None of the genes coding for enzymes of the urea cycle, nopaline or octopine dehydrogenases, nitric oxide synthases, arginine monooxygenases, arginine deiminase, glycine amidotransferase, arginine succinyltransferase, arginine/ornithine decarboxylase and arginine transaminases, have been identified (Figure 2, image 1). These data suggest that *T. cruzi* would be unable to use arginine as an alternative carbon source like proline and other amino acids. To preliminary test this hypothesis different epimastigotes samples were incubated in PBS, or PBS supplemented with 10 mM glucose, proline, glutamate or arginine. After 96 h of treatment motile parasites were observed in proline (92%), glucose (73%) and glutamate (51%), but not in arginine or PBS controls.

Finally, the only enzymes involved in arginine metabolism found in the *T. cruzi* genome project are arginyl-tRNA synthetases and a previously described arginine kinase.

### Arginine transport is related to arginine metabolism

N-Phosphorylated guanidino compounds, commonly referred to as phosphagens, play a critical role as an energy reserve because of the high energy phosphate that can be transferred when the renewal of ATP is needed. It has also been proposed that these compounds function in spatial buffering of cellular energy production sites. So, phosphagens act as reserves not only of ATP but also of inorganic phosphate, which is mostly returned to the medium by metabolic consumption of ATP [20]. Earlier studies demonstrated that *T. cruzi* arginine transport system is coupled to arginine metabolism, specifically to the arginine kinase-mediated synthesis of phosphoarginine, a phosphagen molecule with a critical role as energy reservoir [6], [20], [21]. Arginine kinase expression in *T. cruzi* epimastigotes is precisely regulated by extracellular conditions increasing its levels during the parasites growth curve [22]. As demonstrated above free intracellular arginine is only used by arginine kinase, in addition to protein synthesis, to synthesize phosphoarginine from ATP. In consequence, the increase in the arginine concentration produced by TcAAP3 over-expression would produce a displacement in the equilibrium of the reaction toward the formation of phosphoarginine with the consequent decrease of the concentration of ATP. Such a dramatic effect as the decrease in ATP probably has compensatory mechanisms. In order to test if this regulatory mechanism is associated to arginine transport, arginine kinase expression was evaluated during the parasite growth curve, in TcAAP3 epimastigotes and GFP controls. Figure 2 (image 2) shows a graphical comparison, of arginine kinase expression, between the parasites that have increased the arginine transport activity (AAP3) and controls (GFP). Western Blot and densitometry analysis demonstrated that arginine kinase expression is significantly lower, up to 32% in day 6^th^, in the parasites that arginine transport is exacerbated. A possible explanation of this down-regulation of arginine kinase is the metabolic need to counteract the displacement of equilibrium toward the formation of phosphoarginine.

### Arginine uptake affects the intracellular ATP levels when glucose is depleted in the medium

**Figure 3:**
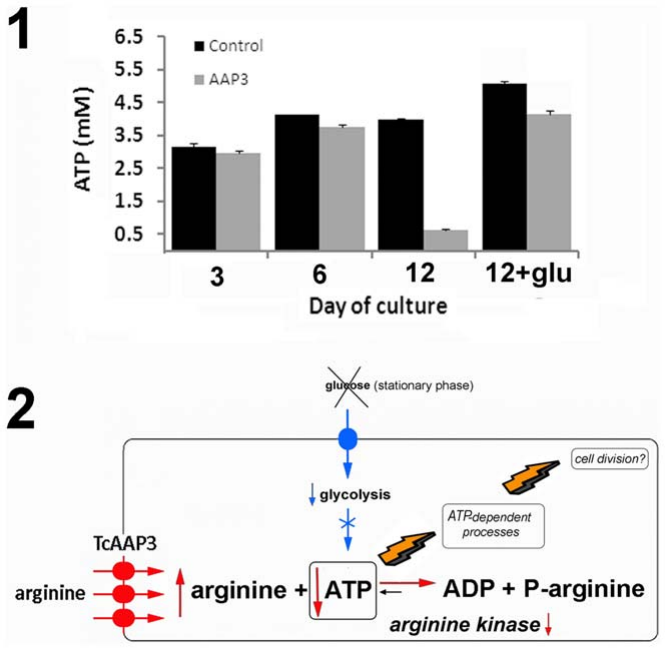
Regulation of arginine metabolism and ATP levels by TcAAP3. 1) Intracellular ATP concentrations were measured using a bioluminescence method and samples from different days of the parasite growth curve. Bars indicated as “12+glu” represent the ATP levels of parasites samples from day 12, cultured overnight in the presence of 2 g.L^−1^ glucose. 2) A brief summary of the arginine transport and metabolism regulation model at the stationary phase of epimastigote growth was constructed from the results herein presented.

In order to test if variations in arginine concentrations could regulate the cell energy homeostasis throughout arginine kinase, the intracellular ATP levels, and the glucose concentration in the media were determined in TcAAP3 epimastigotes and controls samples. If the higher intracellular arginine concentrations affect the arginine kinase equilibrium, the glycolytic ATP synthesis would be a critical step to maintain the cell energy balance. Therefore, according to this model when the extracellular glucose is scarce the ATP levels should decrease more in TcAAP3 epimastigotes. As expected, the measured intracellular ATP levels remained constant between 3 and 4 mM during the first 6 days of the logarithmic phase of growth, and glucose was gradually consumed decreasing from 7 to 2.4 mM. However, in the stationary phase of growth (day 12) when de glucose concentration in the medium is low (<0.5 mM) the ATP levels fall dramatically to 0.6 mM in TcAAP3 epimastigotes but remain constant in controls (Figure 3, image 1). These results indicate that an increase in arginine concentration caused by TcAAP3 over-expression could produce pleiotropic effects on the metabolism of the parasite generated by an imbalance of ATP concentration. Such decrease of ATP concentration could be the cause of the observed lower growth rates of TcAAP3 epimastigotes in the stationary phase. Figure 3 (image 2) summarizes the proposed arginine transport and metabolism regulation model. To test this model, experiments were repeated by adding glucose (2 g.L^−1^) to stationary phase cultures. According to the model's prediction, both, controls and TcAAP3 epimastigotes recovered the intracellular ATP levels (4.8 and 4.0 mM, respectively) after the addition of glucose (Figure 3, image 1, bars indicated as “12+glu”).

### Arginine and lysine transporters are located into the flagellar pocket

**Figure 4:**
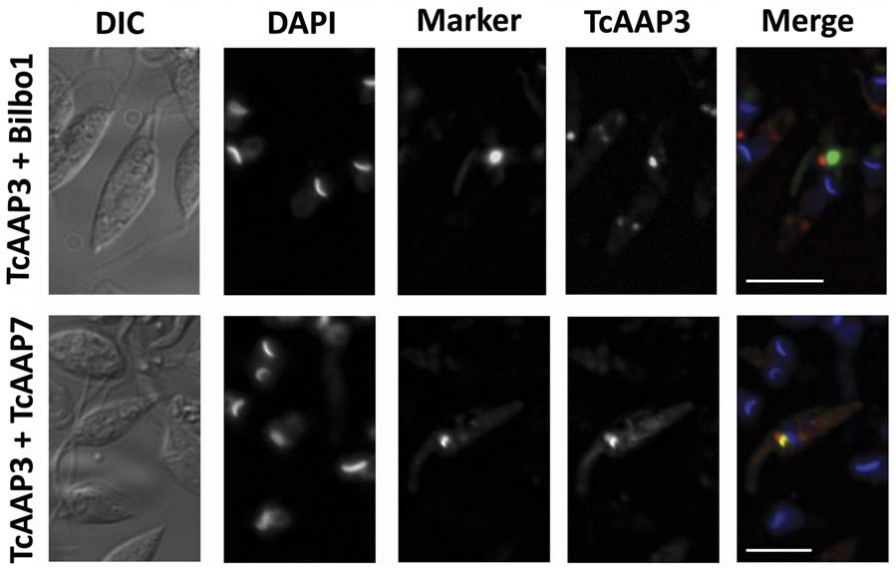
Subcellular localization of TcAAP3. Parasites co-transfected with the pTREXL-mCherry::TcAAP3 and pTREXL-Bilbo1::eGFP (upper panels) or pTREXL-eGFP::TcAAP7 (lower panels) were analyzed by fluorescence microscopy, 24 h post-transfection. Differential interference contrast (DIC), DAPI, eGFP (Marker), mCherry (TcAAP3) and the merged image (Merge) are showed in the indicated columns. White bars in the left corner of the merged images represent 5 µm.

The flagellar pocket is an invagination in the trypanosomatids plasma membrane that constitutes a multiorganelle complex that is involved in the exchange with the extracellular medium and the cell polarity and division [23]. In a previous work we reported that the lysine transporter TcAAP7 was localized mainly in an unidentified membrane structure close to the kinetoplast that could be the flagellar pocket, the cytostome or the contractile vacuole [11]. To determine if TcAAP3 has a similar localization, different plasmid constructions were made using the *T. cruzi* expression vector pTREXL. TcAAP3 (mCherry::TcAAP3) was co-expressed with TcBilbo1 (TcBilbo1::eGFP) or TcAAP7 (eGFP::TcAAP7) in *T. cruzi* epimastigotes. TcBilbo1 was used as a marker of the flagellar pocket's collar, corresponding to a boundary in the flagellar pocket that demarcates a subdomain structure [23], [24]. After parasites transfection, TcAAP3 fluorescence was mainly localized in the flagellar pocket in a single focus close to collar stained by TcBilbo1 (Figure 4). In addition, TcAAP3 co-localized with the lysine transporter TcAAP7 suggesting that the TcAAAP family could be concentrated in a unique region of interchange with the extracellular medium, the flagellar pocket. An interesting observation was the failure in obtaining stable transfectant parasites expressing TcAAP3 fused to a fluorescence protein, suggesting that the fusion protein could act as a dominant negative mutation. In addition, the low TcAAP3 over-expression levels achieved, as mentioned above, reinforce the idea that the precise regulation of intracellular arginine levels is critical for parasite survival. Another issue to highlight is the validation of TcBilbo1 as a flagellar pocket protein, because it is the first marker for this subcellular structure in *T. cruzi*.

*T. cruzi* amino acid transporters translocate their substrates against a concentration gradient that can reach difference of about two orders of magnitude between the mammalian host plasma and the parasite cytosol. For example, arginine concentration reference values of human plasma are between 13–64 µM in adults while *T. cruzi* intracellular concentration is in the millimolar range. These features suggest that amino acid transporters could be used as a gateway for traditional anti-parasitic drugs by the addition of suitable functional groups recognized by these permeases [25] or by rational design of molecules that may alter the transport activity and thus the parasite viability as occurs with TcAAP3.
